# Influence of Microsprinkler Irrigation Amount on Water, Soil, and pH Profiles in a Coastal Saline Soil

**DOI:** 10.1155/2014/279895

**Published:** 2014-07-23

**Authors:** Linlin Chu, Yaohu Kang, Shuqin Wan

**Affiliations:** ^1^National Research Center of Pumps, Jiangsu University, Zhenjiang 212013, China; ^2^Key Laboratory of Water Cycle and Related Land Surface Processes, Institute of Geographic Science and Natural Resources Research, Chinese Academy of Sciences, Beijing 100101, China

## Abstract

Microsprinkler irrigation is a potential method to alleviate soil salinization. After conducting a homogeneous, highly saline, clayey, and coastal soil from the Bohai Gulf in northern China in a column experiment, the results show that the depth of the wetting front increased as the water amount applied increased, low-salinity and low-SAR enlarged after irrigation and water redistribution, and the soil pH increased with an increase in irrigation amount. We concluded that a water amount of 207 mm could be used to reclaim the coastal saline soil in northern China.

## 1. Introduction

The coastal land surrounding Bohai Gulf in northern China is a resource constrained by population growth, rapid industrialization, and agricultural production [1–3]. The soils developed from highly saline coastal mud flats consist of highly saline upper and lower soil horizons, with salinity similar to that of seawater [4]. The shallow groundwater system is also saline, with an electrical conductivity (ECe) between 4.9 and 20.5 dS/m [3, 5]. In addition, strong evaporation increases the rise of salts through the soil profile to the surface. These costal soils are characterized by heavy texture, high content of exchangeable sodium, poor structure, and low water permeability. As a result, the soil does not support vegetation, except for salt resistant halophytes. Therefore, urgent measures are required to alleviate soil salinization and to enable utilization of the saline land.

Several methods have been used to reclaim coastal saline soils including ponding irrigation, chemical amendment, biological reduction, and the replacement of the entire surface soil with nonsaline soil [4, 6–9]. However, these methods are not practical for the coastal land of Bohai Gulf due to the scarcity of freshwater resources and to the low hydraulic conductivities of the soil near saturation. Moreover, when saturated soil conditions persist, growth and production may be inhibited [10, 11]. Chemical amelioration is costly, and developing countries only provide marginal assistance to local farmers for soil amendments. Biological methods for the reduction of salts cannot ameliorate coastal saline soils for long periods of time, and this would be also the case if the entire soil surface would be replaced with a higher quality soil [12–14]. Therefore, there is a need for usable, cheap, and simple methods for combating soil salinization and reclaiming saline land.

Efficient irrigation methods such as drip irrigation, sprinkler irrigation, and microsprinkler irrigation allow precise quantification and positioning of water and chemicals within the soil profile. These methods are considered advantageous due to their high water-use efficiency and their ability to displace salt under unsaturated flow conditions. We have conducted several studies on the efficiency of different drip-irrigation rates for reclaiming coastal saline soils, and we succeeded in the design of adequate reclamation [3, 15].

Sprinkler irrigation, which has some similitude to natural rainfall, has been used to ameliorate saline soils under continuously unsaturated conditions [16, 17]. Nonetheless, the kinetic energy of sprinkler droplets can impact the soil surface, destroy soil aggregates, decrease infiltration rate, and promote leaching. These effects are exacerbated by longer irrigation times and higher irrigation rates [18].

Microsprinkler irrigation has been demonstrated as beneficial for tree and vine crops, because it can promote growth, improve yields, decrease water, and decrease energy use compared to sprinkler or flood irrigation systems, and protects plants from adverse climatic conditions much more than drip irrigation [19]. Microsprinkler irrigation has been examined with a focus on water-use efficiency, crop growth, and system layout, but no studies have focused on the depth distributions of the soil water content (*θ*) and salinity. Drip irrigation is now considered a potential effective method to reclaim the coastal saline soils, but whether microsprinkler irrigation can more efficiently displace salt and more accurately deliver water volumes still remain open questions.

Based on the above rationale, the objectives of this research were to determine the depth distributions of soil water content (*θ*), electrical conductivity (ECe), sodium adsorption ratio (SAR), and pH of saturated soil extracts after application of different water amounts from microsprinklers.

## 2. Materials and Methods

### 2.1. Study Site, Soil Sampling, and Preparation

Soil samples were collected from the Caofeidian Development Zone (39°12′ N; 118°31′ E) in Tangshan Bay Eco-city, east of Hebei province and west of Bohai Gulf. The region has a semihumid marine climate, with a mean annual temperature of 11.4°C and a mean annual precipitation of 554.9 mm, with 74% of the precipitation occurring between June and September. Bulk density, ECe, and pH of the initial soil for each soil layer are shown in Table 1. The average ECe and pH were 29.6 dS/m and 7.4, respectively. In addition to the high ECe, the saturated hydraulic conductivity of the soil (0.0025 mm/d) was very low in fields irrigated with nonsaline water due to soil dispersion and clogging of soil pores.

Soil samples were collected from the A-horizon and were air-dried, crushed, passed through a 2 mm sieve, and characterized for general properties. The ECe, SAR, and pH of the homogenous soils were 27.8 dS/m, 59.7 (mmol/L)^0.5^, and 7.6, respectively. The study soil was classified as a Solonchak and it was silt loam textured, with 0.49% of particles less than 0.002 mm, 45.14% of particles between 0.002 and 0.02 mm, and 54.37% of particles larger than 0.02 mm.

### 2.2. Laboratory Experiment

**Figure 1:**
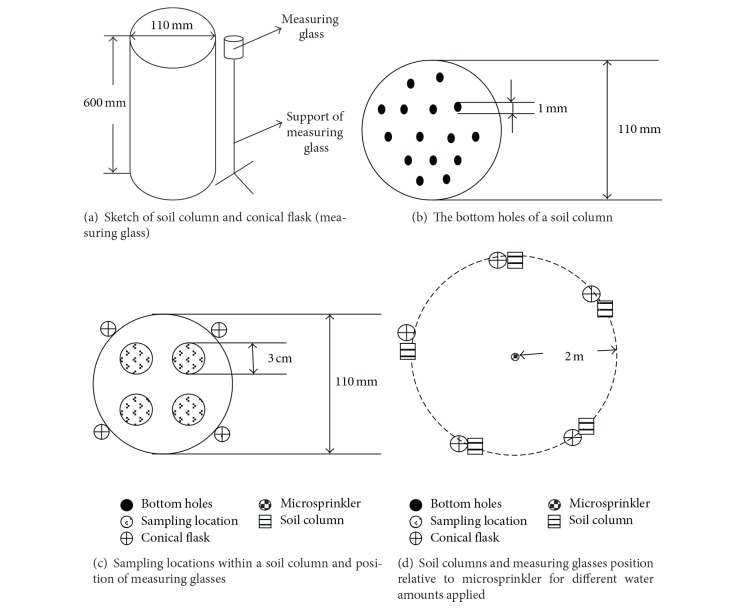
Sketch of the experimental setup showing the location of microsprinkler, soil columns, and measuring glasses, with details of bottom holes and sampling location within a column.

Columns to contain the soil were made of acrylic glass with a diameter and height of 110 mm and 600 mm, respectively, and drainage holes at their base for percolating water (Figure 1(a)). Each soil column was filled with air-dried saline soil to attain 1.5 g/cm^3^, the bulk density of the original soil at the experimental site. The initial soil water content of the experimental columns was 4 gr/100. The soil in the columns was homogeneous and assumed to have vertical water flow throughout the experiment. To reduce the impact of airflow and evaporation, the experiment was conducted in laboratory conditions.

The columns were irrigated using fresh water with an ECe of 0.4 dS/m and a pH of 8.6 via a small plastic swivel microsprinkler (RC-40, Luckrain Irrigation Company, Beijing, China) with a single nozzle, a height of 65 cm, and a wetting radius of 2.5 m. The nozzle operating pressure was maintained at 0.2 MPa using a hydraulic-pressure control valve. The soil columns were all arranged 2 mm apart in a concentric circle with a 2 m radius, and the microsprinkler was positioned at the center (Figure 1(b)). When a column received the designated quantity of water, it was covered immediately with plastic film. Five water amounts (55, 128, 185, 207, and 223 mm) were applied, and each irrigation treatment was repeated three times. The mean air temperature during irrigation was 25°C, and each treatment was irrigated at a rate of 1.2 mm/h.

### 2.3. Measurements of Irrigation Dose and Soil Properties

#### 2.3.1. Irrigation Amounts

The amount of water delivered by the microsprinkler was measured using a conical flask positioned at the same distance from the microsprinkler as the soil columns (Figure 1(c)). After each irrigation event, the quantity of water in each triangular flask was evaluated according to the following:

(1) $$\begin{array}{c} \text{IA}=\frac{m_{2}-m_{1}}{\rho_{w}\times \pi \times r^{2}}\times 10, \end{array}$$

where IA is the irrigation amount (mm); *m*_1_ and *m*_1_ are the quantities of water in the conical flask before and after irrigation was finished, respectively (g); *ρ*_*w*_ is the water density (g/cm^3^); and *r* is the inlet radius of the triangular flask (cm).

#### 2.3.2. Soil Water Content and Salinity

Soil samples for each treatment were extracted vertically from the soil column with an auger (3.0 cm diameter and 100 cm height). Samples were taken during the redistribution period at 0, 24, and 48 h after application of the prescribed water amount was finished (Figure 1(c)). The distance between adjacent vertical samples was set to 1.7 cm (Figure 1(d)). After a full vertical sample was obtained for each period, plastic cloth was placed over the hole to avoid a dehydrating influence of successive samplings. The sample depths were 0–5, 5–10, 10–15, 15–20, 20–25, 25–30, 30–35, 35–40, 40–45, 45–50, 50–55, and 55–60 cm. The water content (*θ*) was determined by oven-drying on a subsample.

For each sampling depth, the remaining soil samples were air-dried and sieved through a 2 mm sieve. A saturated soil paste was prepared via centrifugation (4000 rpm, 20 min) for chemical analysis. The soil ECe, soluble cations (Na^+^, Ca^2+^, and Mg^2+^), and pH were determined in extracts of the saturated soil, and the results were determined using a conductivity meter (DDS-11, REX, Shanghai), inductively coupled plasma optical-emission spectrometer (Optima 5300DV, PerkinElmer, U.S.A), and a pH meter (PHS-3C, REX), respectively. The SAR of the saturated paste extract was calculated using the following:

(2) $$\begin{array}{c} \text{SAR}=\frac{\left[\text{N}{\text{a}}^{+}\right]}{{\left(\left[\text{C}{\text{a}}^{2+}\right]+\left[\text{M}{\text{g}}^{2+}\right]\right)}^{0.5}} \end{array}$$

where the concentration of each cation is in mmol/L.

The average *θ*, ECe, SAR, and pH of the wet zone were calculated as the weighted mean.

#### 2.3.3. Statistical Analysis

Statistical analyses, including a one-way analysis of variance (ANOVA), were conducted to compare and rank the treatment means at the 5% probability level for *θ*, ECe, pH, and periods of soil water redistribution. Statistical analyses were performed using SPSS Version 16.0.

## 3. Results and Discussion 

### 3.1. Wetting Front

When the irrigation time increased from 47 h to 189 h, the irrigation amount increased from 55 mm to 223 mm. This corresponded to a vertical wetting front, just after irrigation has been completed (at 0 h), of 15–20, 40–45, 55–60, 55–60, and 55–60 cm, for each successive irrigation amount. These results showed that as the irrigation amount increased, also the depth of the wetting front at 0 h increased (Table 2). Moreover, the vertical wetting front was not significantly different among 0, 24, and 48 h since irrigation has ceased.

### 3.2. Soil Water Content (*θ*)

**Figure 2:**
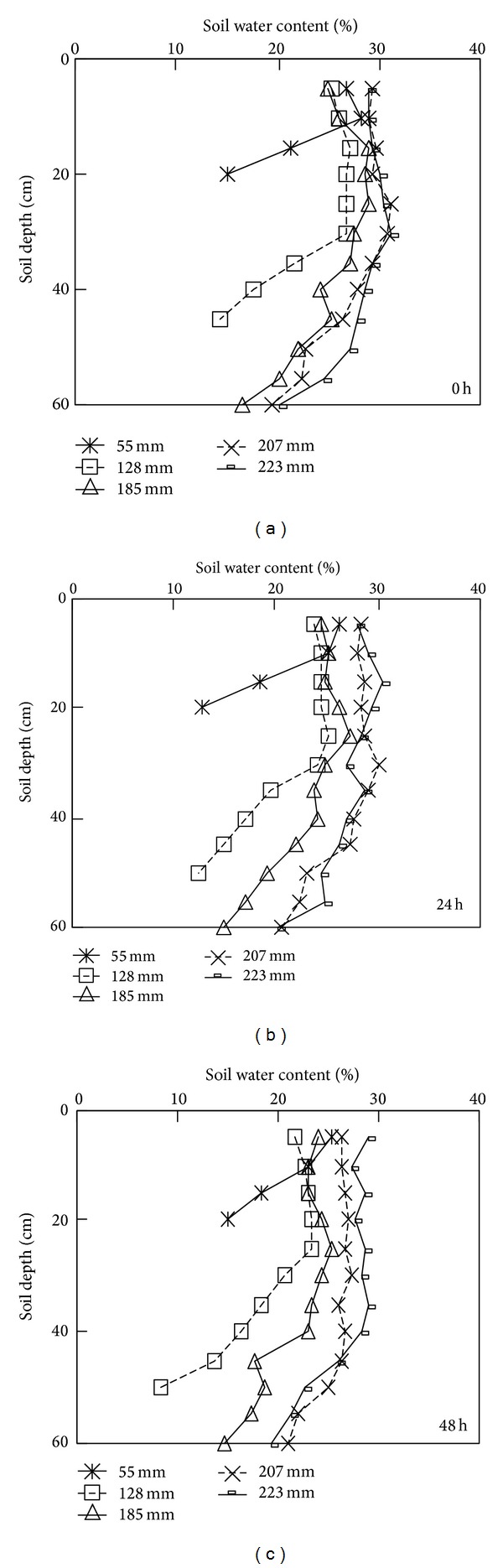
The depth distributions of soil water content at 0 hour (a), 24 hours (b), and 48 hours (c) during water redistribution under different water amounts applied.

The depth distributions of *θ* at 0, 24, and 48 h since irrigation has finished under different irrigation amounts are presented in Figure 2. It is generally accepted that during infiltration *θ* will decrease with increasing soil depth and a relatively moist region forms in the upper layer, where in turn a saturated zone and a transmission zone may be recognized. In our experiment, only a small amount of soil water moved toward the deep layer of the soil column, due to the low hydraulic conductivity of the heavy textured soil studied. Therefore, just after irrigation (0 h), *θ* was greater at 0–20 cm depth than at 20–40 and 40–60 cm for all the studied treatments.

For all the treatments, the water content (*θ*) at the wet upper layer (involving saturation and transmission zones) was uniform and approached the maximum *θ* content. The maximum *θ* values for the irrigation amounts of 55, 128, 185, 207, and 223 mm were 28.3%, 26.9%, 28.8%, 31.1%, and 31.0%, respectively, and were measured at depths of 5–10, 10–15, 10–15, 20–25, and 25–30 cm, respectively. Therefore, as the total irrigation amount was the highest, the depth of the wet zone increased. Furthermore, the average *θ* value of the wet zone showed a trend to increase as the irrigation amount increased. For example, the average *θ* in the wet zone increased from 22.8% (55 mm) to 29.4% (223 mm), and the vertical extent of this zone also increased, which is in accordance with the results of Troiano et al. [20].

As expected, during the redistribution of soil moisture, the matric potential of the soil water showed a trend to approach equilibrium, and consequently the depth differences in *θ* decreased. Compared to the results obtained at 0 h, the water content (*θ*) measured at each depth decreased at 24 h and 48 h after irrigation has ceased. The mean *θ* values of the wet zones decreased by 10.9%, 19.0%, 13.5%, 5.9%, and 5.9% for the irrigation amount of 55, 128, 185, 207, and 223 mm, respectively. Overall, the observed reduction in *θ* decreased with an increase in irrigation amount. During soil-water redistribution events, the air in the soil pore space was replaced by water.

### 3.3. Soil Salinity

#### 3.3.1. Depth Distributions of ECe

**Figure 3:**
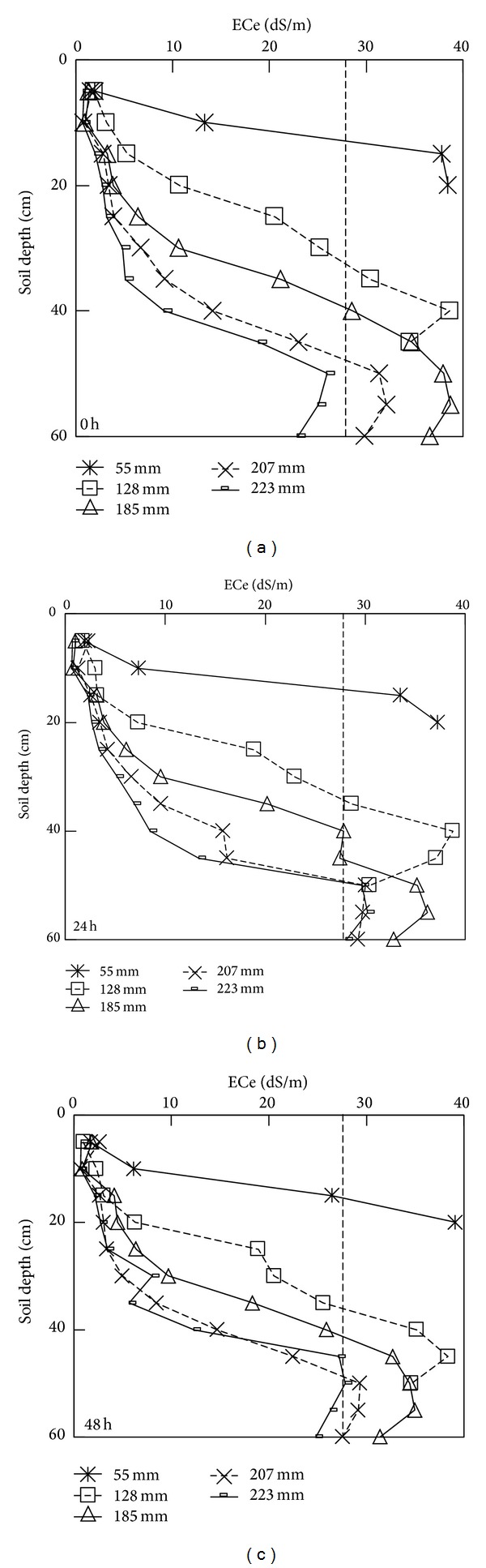
The depth distributions of ECe at 0 hour (a), 24 hours (b), and 48 hours (c) during water redistribution under different water amounts applied.

A consequence of the lateral and vertical movement of water, and the line-source nature of microsprinkler irrigation, is that salts and water are moving toward the subsoil forming a desalinized region at the saturated and transmission zones, and subsequently salts can accumulate near to the wetting front. The profiles of the ECe at 0, 24, and 48 h during water redistribution under different irrigation doses are shown in Figure 3. At 0 h, just after irrigation has ceased, as the irrigation amount increased, also the depth of the layer with low salinity (ECe < 4 dS/m) increased; this layer extended at 0–5, 0–10, 0–20, 0–20, and 0–25 cm for irrigation amounts of 55, 128, 185, 207, and 223 mm, respectively. Besides, the maximum ECe value of the column was significantly higher than the initial value, going up to 38.5, 38.6, 38.7, 30.2, and 32.1 dS/m for irrigation amounts of 55, 128, 185, 207, and 223 mm, respectively. Furthermore, the maximum ECe was observed at a depth of 15–20, 35–40, 50–55, 50–55, and 50–55 cm, for the successive irrigation doses applied. Thus, as the irrigation amount increased, the thickness of the desalinized upper layer increased, while the salt concentration reached a maximum at the middle and lower layers of the column.

During the water redistribution phase, at 48 h, for the irrigation amount of 55 mm, the average ECe decreased as water redistribution progressed, and a 33.8% reduction in ECe was measured at 0–20 cm depth at the 48 h sampling, during water redistribution. Reductions of average ECe for the other four irrigation amounts were not significant, but the desalinization rate of the irrigation amount of 55 mm was significantly lower than those of the other treatments.

#### 3.3.2. Relationship between the Average ECe Value and Irrigation Amount

The relationships between average ECe values in the wetting zone, at successive times during redistribution, and the irrigation amount (IA) can be expressed by the following equation:

(3) average  ECe0=−0.0005(IA)2+0.0614(IA)+20.489 R2=0.8853,average  ECe24=−0.0006(IA)2+0.101(IA)+15.99 R2=0.9614,average  ECe48=−0.0005(IA)2+0.1103(IA)+13.838 R2=0.9216.

**Figure 4:**
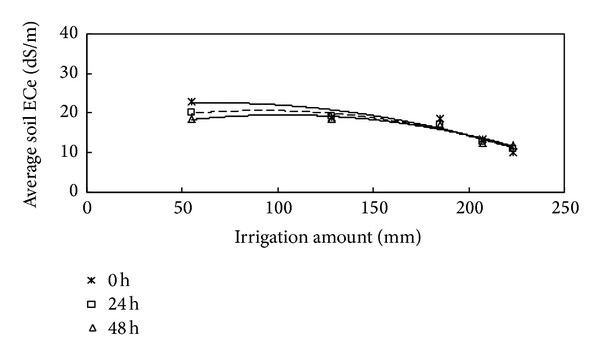
Relationships between average ECe in wetted zone and irrigation amount.

The above relationships show that during redistribution, ECe and irrigation dose could be fitted by parabolic equations with high *R* ^2^ values (Figure 4). Compared to the initial results 0 h after water redistribution, the average ECe values for the wetting zones of irrigation amounts of 55, 128, 185, 207, and 223 mm were decreased by 17.6%, 31.9%, 32.8%, 52.4%, and 63.7%, respectively. When the irrigation amount increased from 55 to 223 mm, the average soil ECe in the wet zone decreased by 55.9% (from 22.9 to 10.1 dS/m).

As the irrigation amount decreased, the average ECe at a depth of 0–20 cm increased by 17.6%, 81.1%, 91.6%, 92.3%, and 94.4%, compared to the initial ECe value. All of the salts were leached down to deeper levels in each irrigation amount, especially for irrigation amounts of 207 and 223 mm. However, the observed differences in the profiles of soil moisture and ECe values among the irrigation amounts of 207 and 223 mm were low during water redistribution. Therefore, a significant change in the average ECe value was not observed as the irrigation amount increased from 207 mm to 223 mm. Meanwhile, the ANOVA results showed that the average ECe for all of the irrigation amounts were significant (*P* < 0.05).

### 3.4. Depth Distribution of SAR

**Figure 5:**
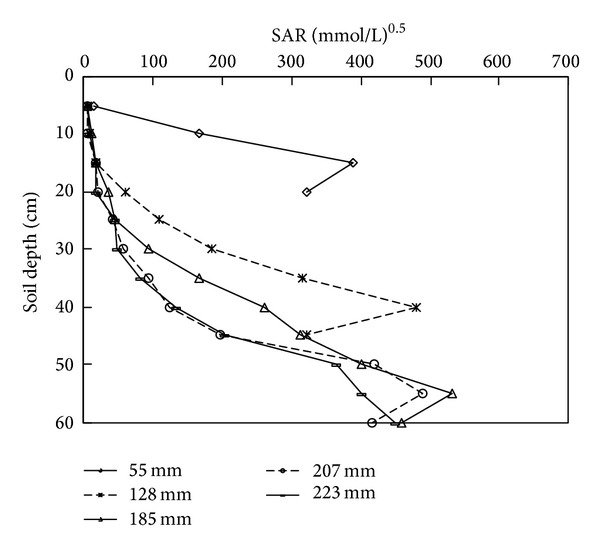
The depth distributions of SAR at 0 hour, just after irrigation has finished, under different water amounts applied.

The sodium adsorption ratio, SAR, is well known to be related to soil dispensability. In practice, SAR and electrical conductivity values of irrigation water allow assessment of its potential to promote soil dispersion. The depth distributions of SAR at 0 h after water application had ceased under the different irrigation amounts are shown in Figure 5. The observed changes in the SAR with successive irrigation doses were similar to those of the ECe values. At the end of the experiment, the SAR of all the treatments increased gradually with an increase in the soil depth.

As the irrigation amount increased, the depth of the highest SAR also increased. For the irrigation amounts of 55, 128, 185, 207, and 223 mm, the zone with the maximum SAR was located at a depth of 10–15, 35–40, 50–55, 50–55, and 55–60 cm, and the corresponding SAR was 387.2, 481.0, 532.6, 489.8, and 450.3 (mmol/L)^0.5^, respectively. However, except for the irrigation amount of 55 mm, a low SAR zone (SAR < 15 mmol/L)^0.5^  was observed at a depth of 0–5 cm. For the other four irrigation amounts, a low SAR zone also was observed at a depth of 0–10 cm. Meanwhile, as the irrigation amount increased, the SAR value of each depth increased, the amplitude of variation increased, and the depth increased, except for the irrigation amounts of 207 and 223 mm. The average soil SAR in the entire profile changed as the experiment progressed. For the irrigation amount of 55 mm, the average soil SAR at a depth of 0–20 cm increased by 272.1%, while that of the other irrigation amounts decreased. A significant reduction in the soil SAR occurred at a depth of 0–20 cm for the irrigation amounts of 128, 185, 207, and 223 mm. Compared to the initial levels, the SAR at 0–20 cm was decreased by 61.3%, 70.4%, 79.2%, and 79.3%, respectively. However, the average SAR at 20–40 and 40–60 cm increased, and the greatest increase occurred at a depth of 40–60 cm. At this depth, the observed increase was more than two times greater than that of the initial value for all four irrigation amounts.

### 3.5. Depth Distribution of Soil pH

**Figure 6:**
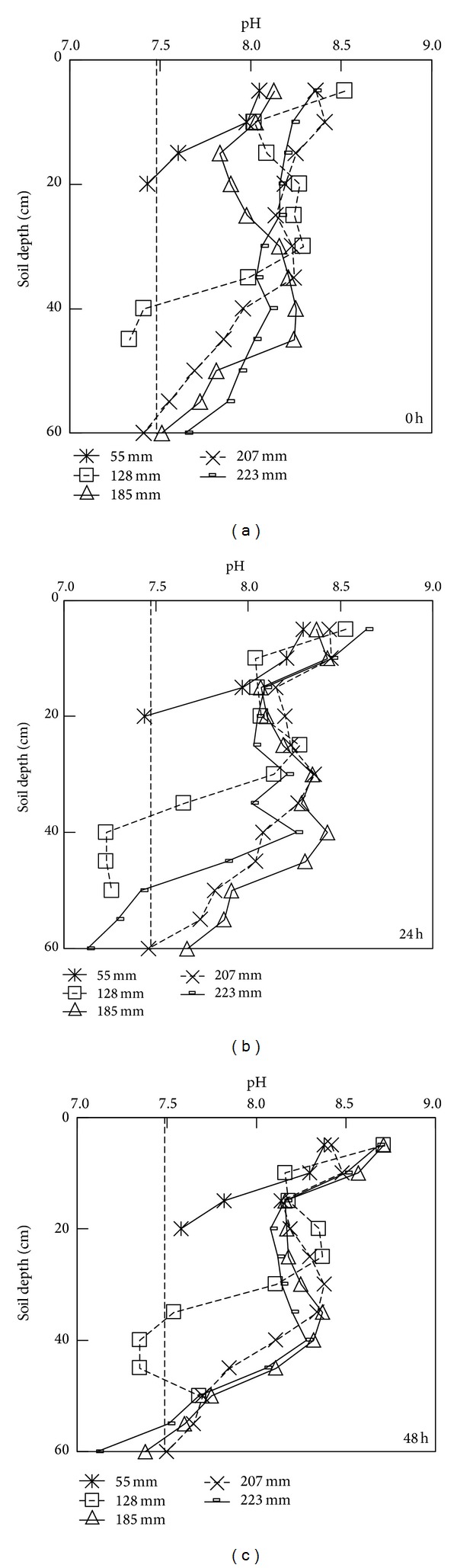
The depth distributions of pH at 0 hour (a), 24 hour (b), and 48 hours (c) during water redistribution under different water amounts applied.

The depth distributions of pH at 0 h, 24 h, and 48 h during water redistribution are shown in Figures 6(a), 6(b), and 6(c), respectively. Changes in the soil pH associated to salt leaching were determined in each irrigation amount. Due to changes in the ECe depth distributions, the profiles of the soil pH in the experimental column changed concomitantly, but the amplitude of its variation was small. For all five irrigation amounts, high soil pH zones were primarily observed in the 0–10 cm layer at the end of irrigation. Moreover, the pH values of profiles obtained for irrigation amounts of 207 and 223 mm were higher than those for 55, 128, and 185 mm. Compared to the initial soil pH, the average pH values of the entire wet zone for the irrigation amounts of 55, 128, 185, 207, and 223 mm were increased by 2.9%, 6.2%, 5.7%, 6.3%, and 6.5%, respectively. Thus, as the irrigation amount increased, the average soil pH also increased, and the irrigation amount of 55 mm significantly differed from each of the other four irrigation amounts.

During soil water redistribution, the layer with high soil pH expanded for each water dose, but the observed differences among successive sampling times in the period of water redistribution were minor. These results indicated that the irrigation amount played an important role in the salt concentration of the soil but had a weaker effect on the average soil pH.

The study focused on the influence of irrigation amounts with microsprinkler irrigation on water, salt, and pH profiles in a coastal saline soil under laboratory conditions. Therefore the outcome of the experiment performed may be affected by some uncertainties. To adapt to the local conditions in northern China, further studies in field should be considered.

## 4. Conclusions

The irrigation amount, which ranged from 55 to 223 mm, had a significant effect on the depth distributions of *θ*, ECe, and SAR but had a relatively minor impact on the depth distributions of pH. Due to the characteristics of the studied soil, mainly the high clay content and low hydraulic conductivity, significant differences between the periods of water redistribution were not observed.

The differences among the depth distributions of *θ* were significant for the irrigation amounts of 55, 128, and 185, but not for those of 207 and 223 mm.

Except for the irrigation amount of 55 mm, salt was leached from the soil, and the low salinity zone enlarged as the irrigation amount increased. Parabolic relationship between the average ECe of the wetting front and the irrigation amounts was observed at 0, 24, and 48 h during water redistribution. Moreover, the reduction in the average ECe ranged from 17.6% for the irrigation amount of 55 mm to 63.7% for the 223 mm treatment.

The depth distributions of the SAR were similar to those of the ECe, but the observed reduction was smaller than that of the ECe.

Taking all of the above factors into consideration, irrigation amounts of 207 mm for 50–60 cm soil depth under microsprinkler irrigation appear to be the best option to reclaim highly saline coastal land surrounding Bohai Gulf in northern China.

## Figures and Tables

**Table 1:** Bulk density, electrical conductivity of saturated paste extracts (ECe), and pH of the initial soil profile sampled at Caofeidian Eco-city, Hebei, China.

Depth (cm)	Bulk density (g/cm^3^)	ECe (dS/m)	pH
0–10	1.32	30.6	7.2
10–20	1.51	29.4	7.3
20–30	1.58	28.8	7.6
30–40	1.71	28.6	7.7
40–60	1.62	28.9	7.5
60–80	1.79	29.6	7.3
80–100	1.62	30.9	7.1
100–120	1.62	30.1	7.1

Average	1.60	29.6	7.4

**Table 2:** Period of irrigation and depth of the wetting front at 0, 24, and 48 h during redistribution for each irrigation amount.

Irrigation amounts (mm)	Irrigation hours (h)	Depth of the wetting front (cm)		
		0 h of water redistribution	24 h of water redistribution	48 h of water redistribution
55	47	15–20	15–20	15–20
128	108	40–45	45–50	45–50
185	156	55–60	55–60	55–60
207	175	55–60	55–60	55–60
223	189	55–60	55–60	55–60
